# Burden of Diagnosed Diabetes among People With and Without HIV in Uganda: a Cross-Sectional Analysis of the Rakai Community Cohort Study

**DOI:** 10.21203/rs.3.rs-9795150/v1

**Published:** 2026-06-28

**Authors:** Rusi Long, Helena Baffoe-Bonnie, Xinyi Feng, Anthony Ndyanabo, Hadijja Nakawooya, Steven J. Reynolds, Ronald Galiwango, Godfrey Kigozi, Thomas C. Quinn, Elizabeth Ogburn, Joseph Kagaayi, Edward Kankaka, M. Kate Grabowski, Robert Ssekubugu, Todd T. Brown, Joseph Ssuuna, Larry William Chang

**Affiliations:** Johns Hopkins University; Johns Hopkins University; Johns Hopkins University; Rakai Health Sciences Program; Rakai Health Sciences Program; Johns Hopkins University; Rakai Health Sciences Program; Rakai Health Sciences Program; Johns Hopkins University; Johns Hopkins University; Rakai Health Sciences Program; Rakai Health Sciences Program; Johns Hopkins University; Rakai Health Sciences Program; Johns Hopkins University; Rakai Health Sciences Program; Johns Hopkins University

**Keywords:** Diabetes, HIV, Sub-Saharan Africa, Non-communicable Diseases, Uganda

## Abstract

The study cohort (N = 17,716) had a mean age of 32.8 years (± 12.4) and was 53.7% female. The weighted prevalence was 0.92% for diagnosed diabetes and 16.9% for lab-confirmed HIV. Females had a higher prevalence of both HIV (20.6%, 95% CI: 19.8–21.4) and diabetes (1.14%, 95% CI: 0.95–1.38%) compared to males. Overall, diagnosed diabetes prevalence did not differ significantly by HIV serostatus (HIV-positive: 0.93%, 95% CI: 0.78–1.08%; HIV-negative: 0.92%, 95% CI: 0.65%−1.32%). After adjusting for age, sex, body mass index (BMI), household-based socio-economic status, and community type, HIV serostatus was not associated with diagnosed diabetes (aPR = 0.75, 95% CI: 0.50–1.13, p = 0.17), even after stratification by sex or community type. Significant independent risk factors for diagnosed diabetes included older age, higher BMI, higher socioeconomic status, and residence in a fishing community.

## INTRODUCTION

Sub-Saharan Africa (SSA) has been undergoing rapid economic growth in recent decades, leading to transformations in disease patterns. While infectious diseases have traditionally dominated attention in the region, recognition of the growing burden of non-communicable diseases, such as diabetes, is increasing. According to the International Diabetes Federation (IDF), persons with diabetes in SSA are projected to reach 59.5 million by 2050, marking a 142% increase from 2024(1). Although global action to combat human immunodeficiency virus (HIV) has had an immense positive impact on SSA (2), multi-morbidity poses a key concern (3, 4).

The association between HIV and diabetes is complex, with an evolving understanding and contradicting evidence (5). The relationship was initially defined in the mid-1990s with the link between specific antiretroviral therapies (i.e. protease inhibitors (6), which are less commonly used today), and adverse metabolic effects. The population-based evidence for an elevated diabetes risk in people living with HIV (PLWH) is largely derived from studies conducted decades ago in Western countries and involving predominantly white participants (7–9). Over time, other concerns emerged, such as the inaccuracy of hemoglobin A1c (HbA1c) in PLWH and potential metabolic risks associated with newer drug classes like integrase inhibitors INSTIs (10). However, at present, most studies in SSA, including both clinic-based (11–15) and population-based (16–19) studies, have not found that the prevalence of diabetes was significantly different based on HIV-related factors such as serostatus, CD4 count, or ART treatment type or duration. Notably, a study in South Africa reported a significantly lower prevalence of Type 2 diabetes among females living with obesity and HIV compared to females living with obesity and without HIV (20). Different impacts of HIV status on diabetes have also been found by age (21) and diabetes care cascade stages (22). This uncertainty may reflect a complex interplay of factors, such as age, differences in immune activation and inflammatory response, differences in health care utilization and access, and referral and diagnostic biases (17, 18).

Understanding the prevalence of diagnosed diabetes in high-burden HIV settings can inform integrated healthcare strategies and highlight disparities in diagnosis and opportunities for improving chronic disease care. The recent IDF report estimated a national prevalence of 2.2% among Ugandans aged 20–79 in 2024, with 61% of cases undiagnosed, based on the nationally representative household-based WHO STEPS 2014 survey and defining diabetes by a single abnormal blood glucose test (1, 23). In SSA, studies examining the relationship between HIV and diabetes have predominantly focused on South Africa (17, 18, 20–22). In Uganda, limited exploration has been done in the general age population. Only one study (16), which focused on the 35–49 age group, investigated the prevalence of diabetes and type 2 diabetes risk and reported no difference in prevalence by HIV serostatus.

The Rakai Community Cohort Study (RCCS) is a well-established, population-based cohort with a historical focus on HIV-related research in southern Uganda since the 1990s. Given the limited data on diabetes prevalence and its association with HIV, we leveraged the RCCS to investigate the association between HIV serostatus and diagnosed diabetes. Our study expands on previous research by including a broader age range (≥ 15 years) and by examining potential effect modification by sex and community type.

## METHODS

### Study setting

The RCCS is an open, population-based cohort in four districts (Maska, Rakai, Lyantonde, Kyotera) in rural Uganda. This cross-sectional study used data from adults aged > = 15 participating in the 20th survey round of RCCS from 1st February 2021 to 25th August 2023. A full description of the RCCS design and data collection procedures has been described in previous literature (24, 25).

#### Primary outcome and exposure variables:

Diagnosed diabetes as the primary outcome was measured by a self-reported question, “Have you ever been told by a doctor or other health workers that you have raised blood sugar or diabetes”. HIV serostatus was tested by a rapid antigen test algorithm with confirmatory immunoassay testing (26). Demographic variables included sex and age, which were categorized into four groups: “15–34 years”, “35–44 years”, “45–54 years”, and “above 55 years old”. Body mass index (BMI) was categorized as < 18.5 kg/m2 for underweight, ≥ 18.5 to < 25 kg/m2 for normal, ≥ 25 to < 30 kg/m2 for overweight, and ≥ 30 kg/m2 for obese (16). Community type was categorized into two types: inland communities, including about 30 agricultural and trading community clusters in and near Rakai District, and 4 fishing communities, which consist of the largest Lake Victoria fishing communities in the Rakai region (26). Household-based socioeconomic status (SES) was assessed by household assets and home construction. To reflect the relative economic standing of a household, it was categorized into four groups: “Lowest”, “Low-middle”, “High-middle”, and “Highest” (27).

### Statistical analysis

Participant characteristics were summarized overall and stratified by HIV serostatus. Continuous variables were described using means and standard deviations (SD) or medians and interquartile ranges (IQR), as appropriate, and categorical variables were summarized using counts and percentages.

Differences between groups were assessed using Student’s *t*-tests for normally distributed continuous variables, Mann–Whitney *U* tests for non-normally distributed continuous variables, and chi-square tests for categorical variables.

We estimated both unweighted and weighted prevalence of self-reported diagnosed diabetes and HIV overall and stratified by age, sex, and community type (fishing vs. inland). Ninety-five percent confidence intervals (CIs) for prevalence estimates were calculated using appropriate methods for binomial proportions(28). Inverse probability weights (IPW) were applied to standardize estimates to the age, sex, and community-type distribution of the underlying Rakai population, thereby accounting for differential participation. These weights were derived based on the probability of inclusion in the analytic sample.

We used Poisson regression with robust variance estimation to estimate prevalence ratios (PRs) and adjusted prevalence ratios (aPRs) with 95% CIs for the association between HIV serostatus and self-reported diagnosed diabetes. This approach was selected to provide interpretable estimates of relative prevalence for a non-rare outcome. Multivariable models adjusted for potential confounders identified a priori, including age, sex, BMI, household SES, and community type. Age and BMI were included as categorical variables based on clinically relevant cut-points. To assess potential effect modification, we conducted stratified analyses by sex and community type. Separate multivariable Poisson regression models were fit within strata, and patterns of association were compared across groups. All analyses were conducted using R version 4.3.2.

## RESULTS

### Characteristics of the Study Population Stratified by HIV Serostatus:

Among 27,630 individuals eligible for the 20th round of RCCS survey, 17,789 participated. Participants were more likely to be older and female as compared to non-participants (Supplemental Table 1). After excluding an additional 73 persons with missing data on HIV serostatus and/or diagnosed diabetes, the final analytical dataset comprised 17,716 participants.

As shown in Table 1, the mean age of study participants was 32.8 ± 12.4 years (Mean ± SD), 53.7% were female, and the majority were residing inland communities (74.4%). Approximately 8.5% were categorized as obese (≥ 30 kg/m2) and 18.8% as overweight (≥ 25 to < 30 kg/m2). Demographic characteristics differed significantly by HIV serostatus.

### Prevalence of HIV Serostatus and Self-reported Diabetes with Stratification by Age, Community Type, and Sex

**Figure 1:**
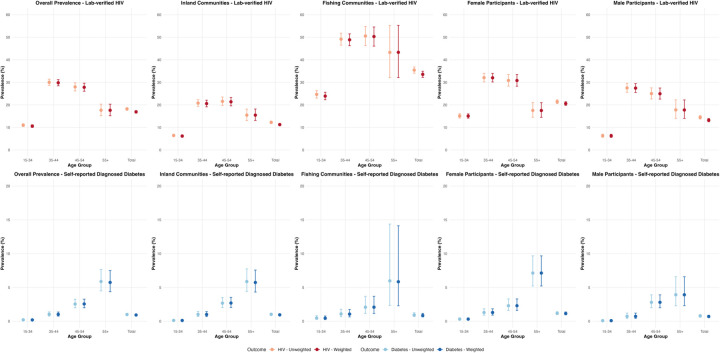
Prevalence of HIV and Diagnosed Diabetes Stratified by Age, Community Type, and Sex (N=17,716). Figure shows prevalence of HIV and diagnosed diabetes with 95% confidence intervals (error bars), stratified by age, sex, and community type. Top panels: HIV prevalence (weighted: red; unweighted: light red). Bottom panels: Diabetes prevalence (weighted: dark blue; unweighted: light blue). Left: Overall estimates. Middle: Stratified by community type (Inland vs. Fishing). Right: Stratified by sex (Female vs. Male). Prevalence estimates were weighted for sampling bias on age, sex, and community type, using inverse probability weights, and the confidence intervals for weighted and unweighted prevalence were based on Wilson score intervals.

Figure 1 illustrates the prevalence of both conditions, with corresponding prevalence values detailed in Supplemental Table 2. Among all 17,716 participants, 176 individuals (unweighted prevalence: 0.99%, 95% CI: 0.86–1.15%; weighted prevalence: 0.92%, 95% CI: 0.79–1.07%) reported a diagnosis of diabetes, while 3,222 participants (unweighted prevalence: 18.19%, 95% CI: 17.63–18.76%; weighted prevalence: 16.90%, 95% CI: 16.36–17.44%) had a positive HIV serostatus. The leftmost panel displays the overall prevalence by age. The age distributions of HIV serostatus and diagnosed diabetes differed; HIV prevalence was highest among middle-aged adults (35–54 years), whereas diabetes prevalence increased steadily with age.

The next two panels of Fig. 1 show the prevalence stratified by age and community type. HIV prevalence was consistently higher across all age groups in the fishing communities compared to the inland communities. In contrast, no significant difference was found in the overall prevalence of diagnosed diabetes by community types (fishing communities = 0.86%, 95% CI: 0.63–1.16%; inland communities = 0.94%, 95% CI: 0.80–1.12%). However, when examined by age group, diabetes prevalence was significantly higher in the fishing communities (0.44, 95%CI: 0.25–0.78) in the 15–34 year age group, compared to 0.11 (95%CI: 0.06–0.22) in inland communities.

The last two panels of Fig. 1 present prevalence stratified by age and sex. Overall weighted prevalence for both HIV and diabetes was higher in female participants: weighted HIV prevalence was 20.57% (95% CI: 19.78–21.39%) in females compared to 13.21% (95% CI: 12.51–13.93%) in males and weighted diabetes prevalence was 1.14% (95% CI: 0.95–1.38%) in females versus 0.70% (95% CI: 0.55–0.89%) in males. This pattern of higher female-weighted prevalence was consistent across all age groups for HIV, although the difference was not statistically significant in the 55 + age group. For diabetes, females also demonstrated a higher weighted prevalence in all age groups except 45–54 year-olds; however, none of these age-specific differences for diabetes reached statistical significance.

### Prevalence of Diagnosed Diabetes by HIV Serostatus, Age, Community Type, and Sex.

**Figure 2:**
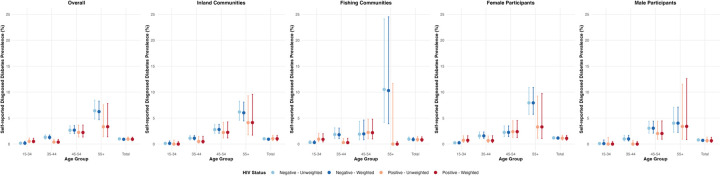
Prevalence of Diagnosed Diabetes by HIV Serostatus, Age, Community Type, and Sex. (N=17,716) Figure shows prevalence of diagnosed diabetes with 95% confidence intervals (error bars), stratified by lab-verified hiv serostatus, age, sex, and community type.. Left: Overall estimates. Middle: Stratified by community type (Inland vs. Fishing). Right: Stratified by sex (Female vs. Male). HIV negative-unweighted: light blue; HIV negative-weighted: dark blue; HIV positive-unweighted: light red; HIV positive-weighted: dark red. Prevalence estimates were weighted for sampling bias on age, sex, and community type, using inverse probability weights, and the weighted confidence intervals were estimated with a logit transformation to ensure prevalence estimates stay within the [0,100%] range for rare events. The confidence intervals for unweighted prevalence were based on Wilson score intervals.

Figure 2 presents the prevalence of diabetes stratified by HIV serostatus, and the prevalence details are reported in Supplement Table 3. The overall weighted diabetes prevalence did not differ significantly by HIV status (HIV-positive: 0.93%, 95% CI: 0.65–1.32%; HIV-negative: 0.92%, 95% CI: 0.74–1.14%). Notably, age-stratified analysis revealed a significant difference in the 35–44 age group, where weighted diabetes prevalence was higher among HIV-negative individuals (1.29%, 95% CI: 0.94–1.76%) than people living with lab-confirmed HIV (0.37%, 95% CI: 0.16–0.89%). When the analysis was further stratified by community type and sex, no significant differences in weighted diabetes prevalence by HIV status were observed overall. A notable exception was found among adults aged 55 and older in fishing communities. In this subgroup, the estimated diabetes prevalence was 10.31% (95% CI: 3.91–24.53%) for HIV-negative individuals, while no cases of diabetes (0.00%, 95% CI: 0.00–0.05%) were reported among individuals with lab-confirmed HIV.

### Association between HIV Serostatus and Diagnosed Diabetes:

Table 2 presents the weighted aPRs for diagnosed diabetes. Diagnosed diabetes was not significantly associated with HIV serostatus in either univariate or multivariate analyses. Instead, age and BMI were the strongest predictors. Compared to the 15–34 age group, prevalence was 4.86 times higher (95% CI: 2.77–8.50, p-value < 0.001) among those aged 35–44, 12.51 times higher (95% CI: 7.38–21.21, p-value < 0.001) among those aged 45–54, and 30.65 times higher (95% CI: 17.99–52.22, p-value < 0.001) among those aged 55 and above. Relative to individuals with a normal BMI, the prevalence was significantly higher among those who were overweight (aPR = 2.69, 95% CI: 1.82–3.99, p-value < 0.001) or obese (aPR = 4.51, 95% CI: 2.86–7.12, p-value < 0.001). We also observed significant associations with SES and community type, with a higher prevalence in the lowest-middle (aPR = 1.85, 95% CI: 1.12–3.08, p-value = 0.014) and highest (aPR = 1.79, 95% CI: 1.15–2.78, p-value = 0.010) household-based SES groups compared to the lowest, and in fishing communities compared to inland communities (aPR = 1.75, 95% CI: 1.10–2.77, p-value = 0.029).

The association between HIV serostatus and diagnosed diabetes was further investigated through stratification by sex and community type. Table 3 reports the modifying effect of sex on the relationship between HIV and diabetes. There was no significant association between HIV serostatus and diagnosed diabetes in either sex (Female: aPR = 0.85, 95% CI: 0.52–1.40, p-value = 0.526; Male: aPR = 0.62, 95% CI: 0.26–1.51, p-value = 0.294). Table 4 shows the relationship between HIV and diabetes stratified by community type. Similarly, no association between HIV serostatus and self-reported diagnosed diabetes was observed in either inland (aPR = 0.77, 95% CI: 0.48–1.22, p-value = 0.257) or fishing communities (aPR = 0.77, 95% CI: 0.25–2.37, p-value = 0.648).

## DISCUSSION

This is one of the first population-based studies from SSA that explores diagnosed diabetes in a high-HIV burden region outside of South Africa (17, 18, 22, 29). In RCCS, where the HIV prevalence is 17%, our study found a low prevalence of diagnosed diabetes (0.92%). Our results align with Uganda's 2016 national report for rural areas (30), which defined diabetes mellitus as fasting capillary whole blood glucose concentration ≥ 7.0 mmol/l or currently on medication for diabetes mellitus and reported 1.0% for rural areas and 2.7% for urban areas. However, it falls below IDF 2024 estimates for Uganda nationally of 1.7% (raw) and 2.2% (age-adjusted), which were based on a single abnormal blood glucose test (1). Among PLWH, diabetes prevalence was 0.90%. While this may in part reflect a genuinely lower underlying prevalence in Uganda compared to, for example, the 9% reported in South Africa using fasting blood glucose tests (31), the figure likely also indicates significant under-detection. This low prevalence, based largely on self-reported data, highlights critical gaps in healthcare access, awareness, and diagnostic capacity in a resource-limited setting where systematic screening is scarce. This aligns with the International Diabetes Federation (IDF) estimates that 61% of diabetes cases in Africa remain undiagnosed (1), as well as the previous studies which indicate that the integration of diagnostics and healthcare for comorbidities of HIV and non-communicable diseases is poor in SSA(4, 32). As non-communicable diseases trend upward across the continent, this low reported prevalence underscores a crucial opportunity for early intervention and improved detection strategies.

Although, HIV infection and antiretroviral therapy (ART) may increase diabetes risk through metabolic and inflammatory pathways (10, 33), the lack of association between HIV and diabetes in our study aligns with previous research conducted in Africa (16–19), where diabetes outcomes were defined using various criteria: Diabetes Risk Scores based on the FINDR ISC model (16, 34), self-reported physician diagnosis (17, 18), use of diabetes medication (17, 18), or meeting standard biochemical thresholds (FPG ≥ 7.0 mmol/L, RPG ≥ 11.1 mmol/L, or HbA1c ≥ 6.5%)(17–19). Of note, almost all PLWH in this study, as in most of Africa and unlike most higher-income settings, were likely on an ART regimen which included tenofovir disoproxil fumarate which can be weight-suppressive. Consistent with previous studies in Africa (17, 18), traditional risk factors, which include older age (17, 18), obesity (16, 17), and higher SES (17, 35), were associated with diagnosed diabetes. We observed a significantly higher prevalence of diagnosed diabetes among females, a finding consistent with prior literature (20). However, although females have a higher proportion of obese and overweight groups, traditional metabolic risks like BMI showed a stronger association with diagnosed diabetes than in males. Among females, socio-environmental factors (residence in fishing communities and higher socioeconomic status) play a crucial role in influencing access to diagnosis and subsequent health behaviors. The finding aligns with previous findings that psychosocial factors have a greater impact on women's diabetes (36), underscoring the need for interventions that address these distinct drivers.

Our findings indicate a dual risk of HIV and diabetes within fishing communities compared to inland communities (37, 38). The weighted HIV prevalence in fishing community is 3 times higher than inland community (Supplement Table 2.). While the weighted prevalence of diagnosed diabetes did not significantly differ between community types (Supplement Table 2), after multivariable adjustment, residing in a fishing community was associated with a 1.75 fold significantly higher prevlaence of diagnosed diabetes compared to inland communities (Table 2). This elevated risk profile may be shaped by socioeconomic and lifestyle factors prevalent in these settings, such as high consumption of alcohol (39, 40) and poor health infrastructure (40), which could influence both HIV and non-communicable disease risk. Furthermore, there are intensified HIV management programs in these areas, leading to more frequent health interactions, which may also enhance the detection of underlying diabetes. Given the high HIV burden in fishing communities (26), integrated diabetes-HIV interventions may be particularly beneficial in this setting. Both diabetes and HIV share comparable characteristics in the health provider, patients, and environment dimensions due to being “chronic conditions”, which highlights potential benefits from care integration and leveraging the experience of prevention and control from HIV to noncommunicable diseases(41).

The study had several limitations. First, the study cannot infer causality due to the cross-sectional nature, and we only assessed the association between HIV and diagnosed diabetes. Secondly, our primary outcome relied on self-reporting, which may have been inaccurate and likely underestimated the prevalence of diabetes due to undiagnosed cases (1, 42). Similarly, our cross-sectional and self-report diabetes design cannot disentangle whether the observed association between HIV and diabetes in fishing communities reflects a true increase in diabetes incidence or heightened detection due to more frequent health system contact. To clarify this distinction, future longitudinal HbA1c or other dysglycemia measurements are needed. Furthermore, previous studies have indicated that ART may have metabolic impacts that increase the risk of diabetes (33). However, due to the availability of data on HIV treatment, we were only able to focus on the HIV serostatus. While we did not have detailed information on the types and duration of ART among our participants, we know that the current ART treatments they received are similar, consisting of one integrase inhibitor (dolutegravir) and two Nucleoside/Nucleotide Reverse Transcriptase Inhibitors (tenofovir disproxil fumarate and lamivudine) and that > 90% of PLWH are on ART in this setting. What’s more, the cut-off point of SES, which was defined before the inclusion of fishing communities in RCCS, may not fully capture the socioeconomic status of fishing community type and may underestimate the impacts of SES on the association between HIV and diabetes in this group (27). Lastly, a study in South Africa reported an increased risk of impaired glucose metabolism during pregnancy for PLWH (29). However, we did not report pregnancy status in our study, considering the uncertainty of the onset time of self-reported diagnosed diabetes.

## CONCLUSION

In summary, the prevalence of diagnosed diabetes was low in rural Uganda, potentially reflecting limited diagnostic access. HIV serostatus was not significantly associated with self-reported diabetes in this analysis, while traditional risk factors as older age and overweight or obese were the primary associated risk factors.. Strategies are needed to better integrate the two diseases in health systems and improve diabetes diagnosis, treatment, and care in rural Uganda and similar settings.

## Supplementary Files

This is a list of supplementary files associated with this preprint. Click to download.

- FinalBMCGPHselfreporteddiabetesSupp20260523.docx

## Figures and Tables

**Table 1:** Demographic Characteristics by HIV Serostatus. (N = 17,716) Notes: BMI: Body Mass Index was categorized as < 18.5 kg/m2 for underweight, ≥ 18.5 to < 25 kg/m2 for normal, ≥ 25 to < 30 kg/m2 for overweight, and ≥ 30 kg/m2 for obese; Household-based SES: Household-based Socioeconomic Status was assessed by household assets and home construction and was categorized into four groups to reflect the relative economic standing of a household.

	HIV-seronegative	HIV- seroppositive	Total	P-value
	(N = 14,494)	(N = 3,222)	(N = 17,716)	
**Age (years)**				
Mean (SD)	31.5 (12.7)	38.3 (9.18)	32.8 (12.4)	p < 0.001
15–34	9078 (62.6%)	1121 (34.8%)	10199 (57.6%)	p < 0.001
35–44	2987 (20.6%)	1280 (39.7%)	4267 (24.1%)	
45–54	1728 (11.9%)	671 (20.8%)	2399 (13.5%)	
55+	701 (4.8%)	150 (4.7%)	851 (4.8%)	
**Sex**				
Female	7477 (51.6%)	2035 (63.2%)	9512 (53.7%)	p < 0.001
Male	7017 (48.4%)	1187 (36.8%)	8204 (46.3%)	
**BMI**				
Normal	9255 (63.9%)	1919 (59.6%)	11174 (63.1%)	p < 0.001
Underweight	1222 (8.4%)	248 (7.7%)	1470 (8.3%)	
Overweight	2656 (18.3%)	680 (21.1%)	3336 (18.8%)	
Obese	1207 (8.3%)	302 (9.4%)	1509 (8.5%)	
Missing	154 (1.1%)	73 (2.3%)	227 (1.3%)	
**Household-based SES**				
Lowest	4734 (32.7%)	1749 (54.3%)	6483 (36.6%)	p < 0.001
Low-middle	2417 (16.7%)	400 (12.4%)	2817 (15.9%)	
High-middle	1425 (9.8%)	346 (10.7%)	1771 (10.0%)	
Highest	5829 (40.2%)	688 (21.4%)	6517 (36.8%)	
Missing	89 (0.6%)	39 (1.2%)	128 (0.7%)	
**Community type**				
Inland	11561 (79.8%)	1613 (50.1%)	13174 (74.4%)	p < 0.001
Fishing	2933 (20.2%)	1609 (49.9%)	4542 (25.6%)	

**Table 2:** Association between HIV Serostatus and Diagnosed Diabetes, N = 17,716.

Variables	Diabetes /Total (%)	Weighted Prevalence (95%CI)	Weighted PR	P-value	Weighted aPR	P-value
**HIV Serostatus**						
Negative	145/14494 (1.00%)	0.92% (0.78%, 1.08%)	Ref		Ref	
Positive	31/3222 (0.96%)	0.93% (0.65%, 1.32%)	1.01 (0.71, 1.44)	0.964	0.75 (0.50, 1.13)	0.171
**Age (years)**						
15–34	21/10199 (0.21%)	0.20% (0.13%, 0.30%)	Ref		Ref	
35–44	44/4267 (1.03%)	1.02% (0.76%, 1.36%)	5.20 (3.12, 8.65)	< 0.001	**4.86 (2.77, 8.50)**	**< 0.001**
45–54	61/2399 (2.54%)	2.55% (1.99%, 3.27%)	13.07 (8.04, 21.26)	< 0.001	**12.51 (7.38, 21.21)**	**< 0.001**
55+	50/851 (5.88%)	5.74% (4.38%, 7.50%)	29.37 (17.43, 49.49)	< 0.001	**30.65 (17.99, 52.22)**	**< 0.001**
**Age (Every 10 years increase)**	/	/	2.12 (2.03, 2.21)	< 0.001	/	
**Sex**						
Female	112/9512 (1.18%)	1.14% (0.95%, 1.38%)	Ref		Ref	
Male	64/8204 (0.78%)	0.70% (0.55%, 0.89%)	0.61 (0.45, 0.82)	0.001	1.08 (0.75, 1.56)	0.715
**Household-based SES**						
Lowest	43/6483 (0.66%)	0.61% (0.45%, 0.82%)	Ref		Ref	
Low-middle	30/2817 (1.06%)	0.87% (0.54%, 1.39%)	1.62 (1.05, 2.52)	0.029	**1.85 (1.12, 3.08)**	**0.014**
High-middle	17/1771 (0.96%)	1.20% (0.97%, 1.49%)	1.42 (0.85, 2.39)	0.18	1.61 (0.91, 2.83)	0.093
Highest	84/6517 (1.29%)	0.99% (0.69%, 1.41%)	1.98 (1.41, 2.78)	< 0.001	**1.79 (1.15, 2.78)**	**0.010**
missing	2/128 (1.56%)	1.51% (0.41%, 5.44%)				
**BMI**						
Normal	58/11174 (0.52%)	0.48% (0.37%, 0.62%)	Ref		Ref	
Underweight	8/1470 (0.54%)	0.48% (0.24%, 0.96%)	1.01 (0.50, 2.02)	0.988	0.85 (0.40, 1.80)	0.687
Overweight	57/3336 (1.71%)	1.66% (1.28%, 2.14%)	3.44 (2.43, 4.86)	< 0.001	**2.69 (1.82, 3.99)**	**< 0.001**
Obese	50/1509 (3.31%)	3.30% (2.51%, 4.33%)	6.85 (4.83, 9.73)	< 0.001	**4.51 (2.86, 7.12)**	**< 0.001**
missing	3/227 (1.32%)	1.29% (0.44%, 3.74%)				
**Community Type**						
Inland	134/13174 (1.02%)	0.94% (0.80%, 1.12%)	Ref		Ref	
Fishing	42/4542 (0.92%)	0.86% (0.63%, 1.16%)	0.91 (0.66, 1.25)	0.552	**1.75 (1.10, 2.77)**	**0.029**

Notes: BMI: Body Mass Index was categorized as < 18.5 kg/m2 for underweight, ≥ 18.5 to < 25 kg/m2 for normal, ≥ 25 to < 30 kg/m2 for overweight, and ≥ 30 kg/m2 for Obese; Household-based SES: Household-based Socioeconomic Status was assessed by household assets and home construction and was categorized into four groups to reflect the relative economic standing of a household. PR: prevalence ratio estimated by univariate poisson regression with robust variance. aPR: adjusted prevalence ratio estimated by multivariate poisson regression with robust variance adjusted for categorical age, sex, household-based SES, BMI and Community type. CI (Confidence Interval). Prevalence estimates were weighted for sampling bias on age, sex, and community type, using inverse probability weights. The unweighted and weighted CIs were estimated by Wilson score intervals.

**Table 3:** Association between HIV Serostatus and Diagnosed Diabetes (Stratified by Sex). Notes: Analysis were stratification by sex group. BMI: Body Mass Index was categorized as < 18.5 kg/m2 for underweight, ≥ 18.5 to < 25 kg/m2 for normal, ≥ 25 to < 30 kg/m2 for overweight, and ≥ 30 kg/m2 for obese; Household-based SES: Household-based Socioeconomic Status was assessed by household assets and home construction and was categorized into four groups to reflect the relative economic standing of a household. PR (prevalence ratio) estimated by univariate poisson regression with robust variance; aPR (adjusted prevalence ratio) estimated by multivariate poisson regression with robust variance adjusted for categorical age, household-based SES, BMI and Community type; CI (Confidence Interval). Prevalence estimates were weighted for sampling bias on age, sex, and community type, using inverse probability weights and the weighted CIs were estimated with logit transformation to ensure prevalence estimates stay within the [0,100%] range for rare events under stratification. The CIs for unweighted prevalence were based on Wilson score intervals.

Variables	Female						Male					
	Diabetes/Total (%)	Weighted Prevalence (95%CI)	Weighted PR	P-value	Weighted aPR	P-value	Diabetes/Total (%)	Weighted Prevalence (95%CI)	Weighted PR	P-value	Weighted aPR	P-value
**HIV serostatus**												
Negative	85/7348 (1.16% [0.94%, 1.43%])	1.15% (0.94%, 1.42%)	Ref		Ref		49/5826 (0.84% [0.64%, 1.11%])	0.71% (0.54%, 0.92%)	Ref		Ref	
Positive	27/2164 (1.25% [0.86%, 1.81%])	1.11% (0.73%, 1.66%)	0.96 (0.62, 1.48)	0.852	0.85 (0.52, 1.40)	0.526	15/2378 (0.63% [0.38%, 1.04%])	0.65% (0.32%, 1.30%)	0.92 (0.47, 1.80)	0.802	0.62 (0.26, 1.51)	0.294
**Age**												
15–34	17/5451 (0.31% [0.19%, 0.50%])	0.31% (0.19%, 0.50%)	Ref		Ref		4/4748 (0.08% [0.03%, 0.22%])	0.08% (0.03%, 0.22%)	Ref		Ref	
35–44	30/2323 (1.29% [0.90%, 1.84%])	1.29% (0.90%, 1.84%)	4.16 (2.29, 7.53)	< 0.001	**3.73 (1.73, 8.04)**	**0.001**	14/1944 (0.72% [0.43%, 1.21%])	0.72% (0.43%, 1.21%)	8.59 (2.69, 27.44)	< 0.001	10.01 (2.06, 48.52)	0.004
45–54	28/1219 (2.30% [1.59%, 3.31%])	2.30% (1.59%, 3.31%)	7.40 (4.11, 13.33)	< 0.001	**6.57 (3.16, 13.67)**	**< 0.001**	33/1180 (2.80% [1.99%, 3.91%])	2.80% (2.00%, 3.91%)	33.35 (11.05, 100.61)	< 0.001	**38.22 (8.58, 170.30)**	**< 0.001**
55+	37/519 (7.13% [5.21%, 9.69%])	7.13% (5.20%, 9.69%)	22.96 (12.65, 41.69)	< 0.001	**24.37 (12.11, 49.08)**	**< 0.001**	13/332 (3.92% [2.29%, 6.63%])	3.92% (2.28%, 6.64%)	46.66 (13.81, 157.74)	< 0.001	**57.35 (12.31, 267.17)**	**< 0.001**
**Age (Every 10 years increase)**	/	/	2.07 (1.95, 2.19)	< 0.001	/		/	/	2.17 (2.04, 2.31)	< 0.001	/	
**Community Type**												
Inland	29/3298 (0.88% [0.61%, 1.26%])	1.12% (0.91%, 1.39%)	Ref		Ref		14/3185 (0.44% [0.26%, 0.74%])	0.75% (0.56%, 0.99%)	Ref		Ref	
Fishing	1/28 (3.57% [0.50%, 21.42%])	1.21% (0.83%, 1.77%)	1.08 (0.71, 1.64)	0.721	**2.13 (1.33, 3.42)**	**0.002**	1/100 (1.00% [0.14%, 6.75%])	0.57% (0.34%, 0.94%)	0.76 (0.45, 1.27)	0.294	1.27 (0.43, 3.73)	0.663
**Household-based SES**												
Lowest	54/3645 (1.48% [1.14%, 1.93%])	0.86% (0.60%, 1.24%)	Ref		Ref		30/2872 (1.04% [0.73%, 1.49%])	0.38% (0.22%, 0.63%)	Ref		Ref	
Low-middle	9/911 (0.99% [0.51%, 1.89%])	1.11% (0.71%, 1.74%)	1.29 (0.73, 2.26)	0.379	1.58 (0.90, 2.77)	0.112	8/860 (0.93% [0.47%, 1.85%])	0.84% (0.46%, 1.53%)	2.24 (1.09, 4.59)	0.027	2.56 (0.83, 7.88)	0.101
High-middle	89/7477 (1.19% [0.97%, 1.46%])	0.89% (0.46%, 1.71%)	1.03 (0.51, 2.06)	0.932	1.37 (0.59, 3.19)	0.467	56/7017 (0.80% [0.61%, 1.04%])	0.84% (0.42%, 1.69%)	2.24 (1.03, 4.87)	0.041	1.98 (0.89, 4.40)	0.093
Highest	23/2035 (1.13% [0.75%, 1.70%])	1.46% (1.12%, 1.90%)	1.69 (1.08, 2.62)	0.021	**1.78 (1.09, 2.92)**	**0.022**	8/1187 (0.67% [0.34%, 1.34%])	0.93% (0.65%, 1.33%)	2.47 (1.42, 4.30)	0.001	1.84 (0.64, 5.29)	0.260
missing	19/1630 (1.17% [0.74%, 1.82%])	2.95% (0.37%, 19.71%)	/		/		11/1187 (0.93% [0.51%, 1.67%])	1.14% (0.16%, 7.79%)	/		/	
**BMI**												
Normal	30/4999 (0.60% [0.42%, 0.86%])	0.59% (0.41%, 0.85%)			Ref		28/6175 (0.45% [0.31%, 0.66%])	0.40% (0.28%, 0.59%)	Ref		Ref	
Underweight	2/505 (0.40% [0.10%, 1.57%])	0.38% (0.09%, 1.53%)	0.65 (0.16, 2.65)	0.546	0.53 (0.10, 2.74)	0.446	6/965 (0.62% [0.28%, 1.38%])	0.53% (0.24%, 1.19%)	1.31 (0.60, 2.87)	0.498	1.12 (0.41, 3.06)	0.826
Overweight	36/2481 (1.45% [1.05%, 2.01%])	1.41% (1.02%, 1.95%)	2.39 (1.48, 3.85)	< 0.001	**1.92 (1.20, 3.08)**	**0.007**	21/855 (2.46% [1.61%, 3.74%])	2.30% (1.50%, 3.52%)	5.69 (3.41, 9.50)	< 0.001	**3.65 (1.99, 6.70)**	**< 0.001**
Obese	41/1376 (2.98% [2.20%, 4.02%])	2.95% (2.17%, 3.99%)	4.99 (3.15, 7.90)	< 0.001	**3.34 (2.01, 5.56)**	**< 0.001**	9/133 (6.77% [3.56%, 12.49%])	6.56% (3.40%, 12.30%)	16.22 (8.41, 31.30)	< 0.001	**7.96 (3.62, 17.52)**	**< 0.001**
Missing	3/151 (1.99% [0.64%, 5.98%])	2.03% (0.64%, 6.19%)			/		0/76 (0.00% [0.00%, 4.74%])	0.00% (0.00%, 0.00%)	/		/	

**Table 4:** Association between HIV Serostatus and Diagnosed Diabetes (Stratified by Community Type)

Variables	Inland Communities						Fishing Communities					
	Diabetes/Total (%)	Weighted Prevalence (95%CI)	Weighted PR	P-value	Weighted aPR	P-value	Diabetes/Total (%)	Weighted Prevalence (95%CI)	Weighted PR	P-value	Weighted aPR	P-value
**HIV serostatus**												
Negative	117/11561 (1.01% [0.84%, 1.21%])	0.93% (0.78%, 1.12%)	Ref		Ref		28/2933 (0.95% [0.66%, 1.38%])	0.87% (0.60%, 1.26%)	Ref		Ref	
Positive	17/1613 (1.05% [0.66%, 1.69%])	1.03% (0.64%, 1.66%)	1.11 (0.69, 1.77)	0.675	0.77 (0.48, 1.22)	0.257	14/1609 (0.87% [0.52%, 1.46%])	0.83% (0.49%, 1.39%)	0.95 (0.53, 1.71)	0.858	0.77 (0.25, 2.37)	0.648
**Age**												
15–34	9/7639 (0.12% [0.06%, 0.23%])	0.11% (0.06%, 0.22%)	Ref		Ref		12/2560 (0.47% [0.27%, 0.82%])	0.44% (0.25%, 0.78%)	Ref		Ref	
35–44	29/2882 (1.01% [0.70%, 1.44%])	1.00% (0.69%, 1.43%)	8.81 (4.15, 18.69)	< 0.001	**7.32 (3.15, 16.98)**	**< 0.001**	15/1385 (1.08% [0.65%, 1.79%])	1.06% (0.64%, 1.75%)	2.38 (1.15, 4.93)	0.02	2.73 (0.72, 10.26)	0.138
45–54	50/1869 (2.68% [2.03%, 3.51%])	2.69% (2.04%, 3.53%)	23.80 (11.59, 48.89)	< 0.001	**19.59 (8.88, 43.20)**	**< 0.001**	11/530 (2.08% [1.15%, 3.71%])	2.08% (1.15%, 3.72%)	4.68 (2.20, 9.97)	< 0.001	**5.62 (1.76, 17.98)**	**0.004**
55+	46/784 (5.87% [4.42%, 7.75%])	5.73% (4.31%, 7.58%)	50.70 (24.12, 106.57)	< 0.001	**46.85 (21.00, 104.57)**	**< 0.001**	4/67 (5.97% [2.26%, 14.85%])	5.84% (2.16%, 14.84%)	13.16 (4.25, 40.69)	< 0.001	**13.89 (2.69, 71.84)**	**0.002**
**Age (Every 10 years increase)**	/	/	2.14 (2.05, 2.23)	< 0.001	/	/	/	/	2.24 (1.88, 2.66)	< 0.001	/	/
Sex												
Female	85/7348 (1.16% [0.94%, 1.43%])	1.12% (0.91%, 1.39%)	Ref		Ref		27/2164 (1.25% [0.86%, 1.81%])	1.21% (0.83%, 1.77%)	Ref		Ref	
Male	49/5826 (0.84% [0.64%, 1.11%])	0.75% (0.56%, 0.99%)	0.67 (0.47, 0.94)	0.019	1.24 (0.76, 2.03)	0.394	15/2378 (0.63% [0.38%, 1.04%])	0.57% (0.34%, 0.94%)	0.47 (0.26, 0.85)	0.013	0.69 (0.29, 1.66)	0.410
**Household-based SES**												
Lowest	17/3284 (0.52% [0.32%, 0.83%])	0.47% (0.29%, 0.76%)	Ref		Ref		26/3199 (0.81% [0.55%, 1.19%])	0.75% (0.51%, 1.10%)	Ref		Ref	
Low-middle	29/2681 (1.08% [0.75%, 1.55%])	1.01% (0.70%, 1.45%)	2.12 (1.21, 3.71)	0.008	**2.14 (1.18, 3.88)**	**0.012**	1/136 (0.74% [0.10%, 5.03%])	0.60% (0.08%, 4.23%)	0.81 (0.15, 4.26)	0.8	0.87 (0.14, 5.66)	0.888
High-middle	11/951 (1.16% [0.64%, 2.08%])	1.00% (0.55%, 1.81%)	2.11 (1.07, 4.19)	0.032	**2.71 (1.38, 5.32)**	**0.004**	6/820 (0.73% [0.33%, 1.62%])	0.70% (0.31%, 1.57%)	0.94 (0.40, 2.23)	0.892	0.93 (0.32, 2.68)	0.893
Highest	76/6228 (1.22% [0.98%, 1.53%])	1.14% (0.91%, 1.43%)	2.41 (1.48, 3.93)	< 0.001	**1.91 (1.12, 3.27)**	**0.018**	8/289 (2.77% [1.39%, 5.44%])	2.50% (1.24%, 4.97%)	3.35 (1.64, 6.84)	0.001	2.51 (0.76, 8.31)	0.131
missing	1/30 (3.33% [0.47%, 20.20%])	2.59% (0.33%, 17.59%)	/	/	/	/	1/98 (1.02% [0.14%, 6.88%])	1.18% (0.16%, 8.06%)	/	/	/	/
**BMI**								1.18% (0.16%, 8.06%)				
Normal	46/8387 (0.55% [0.41%, 0.73%])	0.51% (0.38%, 0.68%)	Ref		Ref		12/2787 (0.43% [0.24%, 0.76%])	0.40% (0.23%, 0.70%)	Ref		Ref	
Underweight	6/1231 (0.49% [0.22%, 1.08%])	0.43% (0.19%, 0.96%)	0.85 (0.38, 1.89)	0.684	0.70 (0.29, 1.73)	0.440	2/239 (0.84% [0.21%, 3.28%])	0.77% (0.19%, 3.08%)	1.95 (0.48, 7.89)	0.351	1.96 (0.34, 11.37)	0.455
Overweight	42/2418 (1.74% [1.29%, 2.34%])	1.70% (1.25%, 2.29%)	3.33 (2.24, 4.95)	< 0.001	**2.49 (1.53, 4.04)**	**< 0.001**	15/918 (1.63% [0.99%, 2.69%])	1.55% (0.93%, 2.57%)	3.89 (1.90, 7.96)	< 0.001	**3.42 (1.33, 8.78)**	**0.010**
Obese	38/1082 (3.51% [2.57%, 4.79%])	3.51% (2.56%, 4.81%)	6.90 (4.64, 10.28)	< 0.001	**4.45 (2.46, 8.03)**	**< 0.001**	12/427 (2.81% [1.60%, 4.88%])	2.76% (1.57%, 4.81%)	6.93 (3.32, 14.48)	< 0.001	**4.72 (1.67, 13.31)**	**0.003**
Missing	2/56 (3.57% [0.89%, 13.19%])	3.28% (0.79%, 12.70%)	/	/	/	/	1/171 (0.58% [0.08%, 4.03%])	0.61% (0.08%, 4.27%)	/	/	/	/

Notes: Analysis were stratification by community type. BMI: Body Mass Index was categorized as < 18.5 kg/m2 for underweight, ≥ 18.5 to < 25 kg/m2 for normal, ≥ 25 to < 30 kg/m2 for overweight, and ≥ 30 kg/m2 for obese; Household-based SES: Household-based Socioeconomic Status was assessed by household assets and home construction and was categorized into four groups to reflect the relative economic standing of a household. PR (prevalence ratio) estimated by univariate poisson regression with robust variance. aPR (adjusted prevalence ratio) estimated by multivariate poisson regression with robust variance adjusted for categorical age, sex, household-based SES, and BMI. CI (Confidence Interval). Prevalence estimates were weighted for sampling bias on age, sex, and community type, using inverse probability weights and the weighted CIs were estimated with logit transformation to ensure prevalence estimates stay within the [0,100%] range for rare events under stratification. The CIs for unweighted prevalence were based on Wilson score intervals.

## Data Availability

The datasets used during the current study are available from the RHSP with a reasonable request.

## Abbreviations

ART: Antiretroviral Therapy
BMI: Body Mass Index
CIs: Confidence Intervals
HIV: Human Immunodeficiency Virus
IDF: International Diabetes Federation
INSTI: Integrase Inhibitor
IPW: Inverse Probability Weights
NRTIs: Nucleoside/Nucleotide Reverse Transcriptase Inhibitors
PLWH: People Living with HIV
PR: Prevalence Ratio
RCCS: Rakai Community Cohort Study
RHSP: Rakai Health Sciences Program
SES: Socioeconomic Status
SSA: Sub-Saharan Africa
